# Detection of methoxylated and hydroxylated polychlorinated biphenyls in sewage sludge in China with evidence for their microbial transformation

**DOI:** 10.1038/srep29782

**Published:** 2016-07-15

**Authors:** Jianteng Sun, Lizhong Zhu, Lili Pan, Zi Wei, Yao Song, Yuduo Zhang, Liping Qu, Yu Zhan

**Affiliations:** 1 Department of Environmental Science, Zhejiang University, Hangzhou, Zhejiang 310058, China; 2 Zhejiang Provincial Key Laboratory of Organic Pollution Process and Control, Hangzhou, Zhejiang 310058, China; 3 Analysis and Measurement Center, Zhejiang University, Hangzhou, Zhejiang 310058, China; 4 THUNIP Holdings Co., Ltd., Beijing 100020, China

## Abstract

The concentrations of methoxylated polychlorinated biphenyls (MeO-PCBs) and hydroxylated polychlorinated biphenyls (OH-PCBs) were measured in the sewage sludge samples collected from twelve wastewater treatment plants in China. Two MeO-PCB congeners, including 3′-MeO-CB-65 and 4′-MeO-CB-101, were detected in three sludge with mean concentrations of 0.58 and 0.52 ng/g dry weight, respectively. OH-PCBs were detected in eight sludge samples, with an average total concentration of 4.2 ng/g dry weight. Furthermore, laboratory exposure was conducted to determine the possible source of OH-PCBs and MeO-PCBs in the sewage sludge, and their metabolism by the microbes. Both 4′-OH-CB-101 and 4′-MeO-CB-101 were detected as metabolites of CB-101 at a limited conversion rate after 5 days. Importantly, microbial interconversion between OH-PCBs and MeO-PCBs was observed in sewage sludge. Demethylation of MeO-PCBs was favored over methylation of OH-PCBs. The abundant and diverse microbes in sludge play a key role in the transformation processes of the PCB analogues. To our knowledge, this is the first report on MeO-PCBs in environmental matrices and on OH-PCBs in sewage sludge. The findings are important to understand the environmental fate of PCBs.

Polychlorinated biphenyls (PCBs) are ubiquitous and persistent organic pollutants that are toxic to biota and humans^1^. Once released into the environment, PCBs are susceptible to a variety of transformation pathways^2^,^3^. With increasing scientific interest, the hydroxylated metabolites of PCBs (OH-PCBs) have been considered as environmental contaminants and detected in various media, including biotic samples (peregrine falcons, snapping turtles, polar bears, and bowhead whales)^4^,^5^,^6^,^7^ and abiotic samples (air, surface water, precipitation and sediment)^8^,^9^,^10^. Furthermore, OH-PCBs in human blood reached a level comparable to PCBs^11^,^12^. *In vivo* and *in vitro* studies using animal and plant models have confirmed the transformation from PCBs to OH-PCBs^13^,^14^,^15^,^16^. OH-PCBs are generally considered to form via oxidative mechanisms, such as epoxide intermediates and the direct insertion of the hydroxyl group into a biphenyl, which is mediated by cytochrome P450 enzymes^17^,^18^. For most toxicological endpoints, OH-PCBs are more toxic than their parent PCBs, which affect the endocrine system, brain development, and reproductive processes^19^,^20^.

Recently, we reported the formation of methoxylated metabolites of PCBs (MeO-PCBs) in intact rice plants via laboratory exposure^21^. The interconversion between OH-PCBs and MeO-PCBs was also observed. These results suggested that the generation of MeO-PCBs and their interconversion with OH-PCBs are important metabolic pathways that nevertheless have been ignored. To date, there is no information regarding the occurrence of MeO-PCBs in the environment. Their chemical structures suggest that MeO-PCBs are possibly more lipophilic and persistent than OH-PCBs. Thus, it is important to determine the presence and behavior of newly identified MeO-PCBs other than OH-PCBs in the environment.

Previous studies have reported that a variety of organic pollutants, such as PCBs and polybrominated diphenyl ethers (PBDE)^22^,^23^, have been detected at relatively high concentrations in sewage sludge from wastewater treatment plants (WWTPs), indicating WWTPs are important sinks and secondary emission sources of these chemicals to the ambient environment^24^,^25^. There are currently no published data on the levels of OH-PCBs and MeO-PCBs in sewage sludge. A previous study supposed that OH-PCBs in surface waters that were collected near WWTPs might be the result of microbial oxidation or other oxidation treatment processes^9^, which however, has not been verified. Natural microbes play an important role in the biotransformation of various pollutants^26^,^27^. The hydroxylation of PCBs is a step in the PCB degradation process of white-rot fungi^28^. Various bacterial groups are capable of degrading PCBs via aerobic oxidative processes and anaerobic reductive processes^29^,^30^. As a complex matrix, sewage sludge contains a large number of microorganisms that can cause diverse transformation of PCBs^31^. Sewage sludge may be used to track the presence and fate of MeO-PCBs and OH-PCBs on a large geographical scale.

In the present work, sewage sludge samples were collected from 12 cities in China to provide valuable information on the presence, distribution, and potential sources of MeO-PCBs and OH-PCBs in the environment. We further investigated the microbial transformation of PCB, MeO-PCB, and OH-PCB congeners in a laboratory-simulated environment. This study reported the presence of MeO-PCBs in the environment, and proposed possible microbial transformation between MeO-PCBs and OH-PCBs in sewage sludge.

## Results and Discussion

### Concentrations and compositions of MeO-PCBs and OH-PCBs in sludge

**Figure 1:**
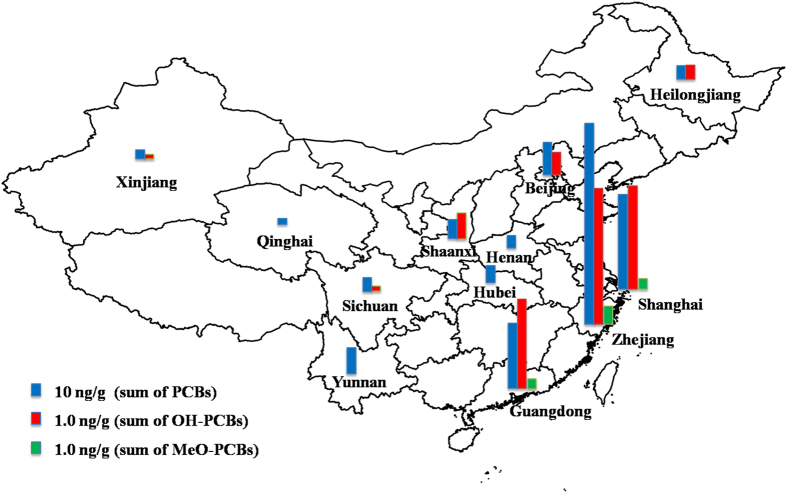
Sampling locations and spatial distributions of the concentrations of PCBs, OH-PCBs, and MeO-PCBs in the sewage sludge samples from twelve wastewater treatment plants in Greater China. The map was produced on ArcGIS 10.3.1 (http://www.esri.com/).

The measured concentrations of the targeted analytes in the sewage sludge are summarized in Table 1. The spatial distributions of ΣPCBs, ΣOH-PCBs, and ΣMeO-PCBs in the sewage sludge from different sampling locations in Greater China are shown in Fig. 1. In this study, two MeO-PCB congeners, 3′-MeO-CB-65 and 4′-MeO-CB-101, were detected in the sewage sludge samples from WWTPs in Zhejiang Province, Guangdong Province, and Shanghai Municipality. The concentrations ranges of 3′-MeO-CB-65 and 4′-MeO-CB-101 were 0.41–0.89 ng/g and 0.43–0.65 ng/g, respectively.

OH-PCBs were found in 8 sludge samples with a detection rate of 67%. Four OH-PCB congeners, including 3′-OH-CB-65, 4′-OH-CB-101, 4′-OH-CB-18, and 4′-OH-CB-26, were identified. The total concentrations of the OH-PCBs ranged from <0.1 to 11.5 ng/g, with a mean of 4.23 ng/g. The dominant congeners were 3′-OH-CB-65 (mean 49% of the total concentration of OH-PCBs) and 4′-OH-CB-101 (mean 33% of the total concentration of OH-PCBs). The number of reports on the occurrence of OH-PCBs in abiotic samples is limited. The detected concentrations of OH-PCBs in the sludge of the present study were comparable to those detected in the sediment from Lake Michigan, USA (0.20 to 26 ng/g with a mean of 8.5 ng/g)^10^.

To investigate the relationships among PCBs, OH-PCBs, and MeO-PCBs, the contamination status of the PCBs in the sludge samples was determined. The total concentrations of the PCBs in the sludge samples ranged from 3.0 to 170 ng/g with a mean of 35.8 ng/g and a detection rate of 100%. The dominant PCBs were CB-28, 52, and 101, which were commonly used to indicate the PCB contamination in the environment^32^,^33^. The results show that low-chlorinated PCBs are the major PCB homologue group residing in sewage sludge in Chinese WWTPs. There were also several peaks of unknown compounds detected in the chromatograms, which might be the metabolites of CB-28, CB-52 or other PCBs, though none of them were identified due to the lack of authentic standards at the time of analysis. This study focused on only ten low-chlorinated OH-PCBs that were found in sediment and commercial PCB mixtures, together with ten homologous MeO-PCBs. More research is needed to identify more potential OH-PCBs and MeO-PCBs in the ambient environment.

The PCBs, OH-PCBs, and MeO-PCBs showed higher levels in Zhejiang Province, Shanghai Municipality, and Guangdong Province. The concentration of PCBs in the surface soil of Shanghai Municipality was found higher than other regions of China^34^. In Zhejiang and Guangdong Provinces, electronic waste recycling activities are considered a very important emission source of PCBs^35^,^36^. The concentrations of the ΣPCBs were significantly correlated with those of the ΣOH-PCBs (*R* = 0.755, *p* < 0.01) and ΣMeO-PCBs (*R* = 0.762, *p* < 0.01). The concentrations of the ΣOH-PCBs were also significantly correlated with those of the ΣMeO-PCBs (*R* = 0.776, *p* < 0.01). For the individual homologous congeners, the concentrations of 3′-OH-CB-65 were significantly correlated with those of 3′-MeO-CB-65 (*R* = 0.791, *p* < 0.05). Close correlations were found among 4′-OH-CB-101, 4′-MeO-CB-101 and CB-101 (*R* = 0.762–0.839, *p* < 0.01). The concentrations of high chlorinated CB-153 were also significantly correlated with those of low chlorinated 4′-OH-PCB-101 and 4′-MeO-PCB-101 (*R* = 0.895–0.946, *p* < 0.01). A strong correlation between concentrations of two contaminants may suggest transformation relationships and/or common sources. No significant relationship was observed between the concentrations of OH-PCB and the total organic carbon (TOC) content, or between the concentrations of MeO-PCBs and the TOC content in the sludge samples (*p* > 0.05).

The highest levels of the three compound groups were all in the WWTP from Zhejiang Province. This WWTP, which was located near an electronic waste dismantling area, treated a mixture of domestic and industrial wastewater. Influent and effluent samples in this WWTP were analyzed to explore the possible source of the analytes (i.e., OH-PCBs and MeO-PCBs). PCBs were detected in the suspended particulate matter (SPM) of the influent and effluent at concentrations of 6.9 ng/g and 1.3 ng/g, respectively. PCBs were also found in water of the influent and effluent with concentrations of 679 pg/L and 141 pg/L, respectively. The concentrations of PCBs in the sludge were higher than those in the wastewater. Only one OH-PCB congener, i.e., 3′-OH-CB-65, was identified in the influent, with concentrations of 0.9 ng/g in the SPM and 65 pg/L in the water. MeO-PCBs were not found in the influent and effluent samples, corroborating their formation in sludge.

The hypothetical precursor of 3′-OH-CB-65 and 3′-MeO-CB-65, namely CB-65, was not found in the sludge or wastewater, suggesting that 3′-OH-CB-65 and 3′-MeO-CB-65 might not be formed as the metabolite of CB-65 in the wastewater treatment process. A previous study reported the presence of several OH-PCBs in the original Aroclors, and found 3′-OH-PCB-65 to be the most prominent congener in Aroclors 1221, 1242, 1248, and 1254^10^. This indicated that the accumulation of 3′-OH-CB-65 in sewage sludge was at least partially due to OH-PCB contamination of the original Aroclors. Although PCBs have been banned for use, there was still considerable emission from the disposal of PCB-containing materials^37^. Therefore, WWTPs were possible receivers of PCBs and the coexisting OH-PCBs. The persistence of 3′-OH-CB-65 should also be concerned since it was recently detected in the sediment of the Lake Michigan. 4′-OH-CB-101, 4′-MeO-CB-101, and their parent compound, CB-101, were all detected in the sludge samples. In our previous study where rice plants were used as the model, CB-61 was biotransformed to 4′-OH-CB-61 (major metabolite) and 4′-MeO-CB-61 (minor metabolite)^21^. Moreover, the interconversion between OH-PCBs and MeO-PCBs is an important metabolic pathway. On the basis of these observations, we hypothesized that microbes in sludge play a key role in the formation of OH-PCBs and MeO-PCBs. The results of the exposure study provide compelling evidence for this hypothesis.

### Metabolism of PCBs, MeO-PCBs and OH-PCBs by microbes in sludge

**Figure 2:**
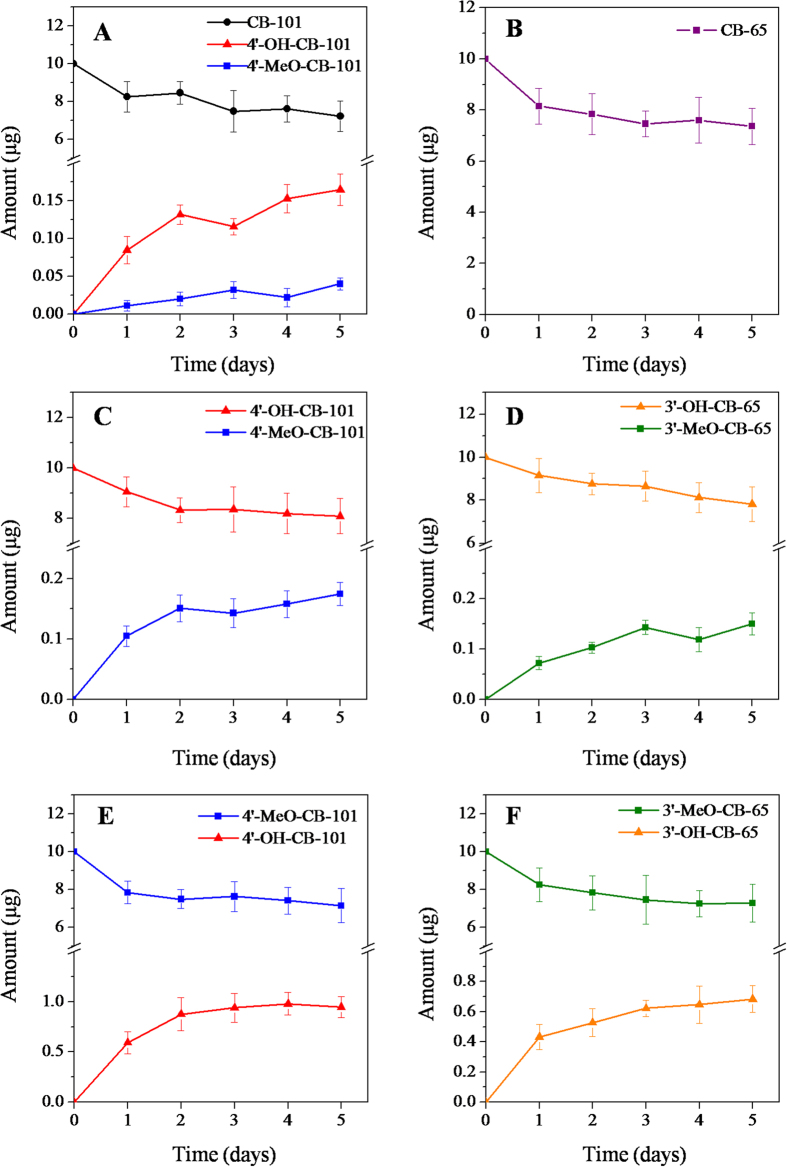
Amounts of exposure compounds and their metabolites in the sludge exposed to (**A**) CB-101, (**B**) CB-65, (**C**) 4′-OH-CB-101, (**D**) 3′-OH-CB-65, (**E**) 4′-MeO-CB-101, and **(F)** 3′-MeO-CB-65 during the 5-day study period.

The hydroxylated and methoxylated metabolites of CB-101 and CB-65 in exposed sludge were analyzed. The 4′-OH-CB-101 and 4′-MeO-CB-101 were identified after the sludge was exposed to CB-101, whereas 3′-OH-PCB-65 and 3′-MeO-PCB-65 were not detected as metabolites of CB-65 (Fig. 2). The metabolic properties of PCBs may depend on the number and location of chlorine atoms on the ring structure of the PCB molecule^38^. Moreover, the hydroxyl and methoxyl were likely to preferentially occur at the *para* position, which needs the least energy during enzymatic reaction^39^.

The interconversion between OH-PCBs and the related MeO-PCBs mediated by microbes in sludge was observed. Transformations from OH-PCBs to MeO-PCBs and from MeO-PCBs to OH-PCBs both occurred after sludge exposure (Fig. 2). The results were consistent with those of the plant exposure study, further supporting that the recently found metabolic pathway of PCBs is ubiquitous and may reflect real PCB biotransformation in the environment^21^. Moreover, similar hydroxylation and methoxylation pathways of PBDEs in plants and animals have been proposed in previous studies^40^,^41^,^42^. The comprehensive results show that a reciprocal transformation between hydroxylated and methoxylated metabolites of other compounds may also exist in the environment.

The conversion percentages, the mass of the metabolite after 5-day exposure over that of the initial parent compound (M/P), are shown in Table 2. The average M/P values were 1.65% for 4′-OH-CB-101/CB-101 and, 0.40% for 4′-MeO-CB-101/CB-101. The transformation rate of CB-101 in sludge was greater than that of CB-61 in rice plant^21^. The average M/P values were 1.51% for 3′-MeO-PCB-65/3′-OH-PCB-65, and 6.83% for 3′-OH-PCB-65/3′-MeO-PCB-65. The average M/P values were 1.75% for 4′-MeO-CB-101/4′-OH-CB-101, and 9.47% for 4′-OH-CB-101/4′-MeO-CB-101. Overall, the demethylation of MeO-PCBs was favored over the methylation of OH-PCBs. Consequently, the concentrations and detection rates of OH-PCBs were higher than those of MeO-PCBs in the collected sludge samples. This may also explain why OH-PCBs have been widely detected, whereas MeO-PCBs have never been observed in the environment. There might be co-elution of isomers in the analysis of sludge samples from WWTPs, which could not be completely avoided due to the large number of isomers and lack of authentic standards. However, the results of these exposure experiments, from some point, verified the detection of targeted compounds.

All the transformation processes occurred rapidly in the sludge, and the metabolites were detected after exposure for only one day (Fig. 2). Generally, the mean residual amount of exposure compounds in sludge slightly decreased, and the mean amount of metabolites gradually increased over 5 days, though no statistically significant difference between the different time points was observed (*p* > 0.05). The metabolism between OH-PCBs and MeO-PCBs may occur simultaneously in the sludge. The mean recoveries of these compounds ranged from 74% to 83% for the exposure groups. This indicated that these compounds may be utilized by microbes in sludge as carbon sources. Some other unknown metabolites, such as diOH-PCBs may also be generated, though none of them have been identified^30^.

**Figure 3:**
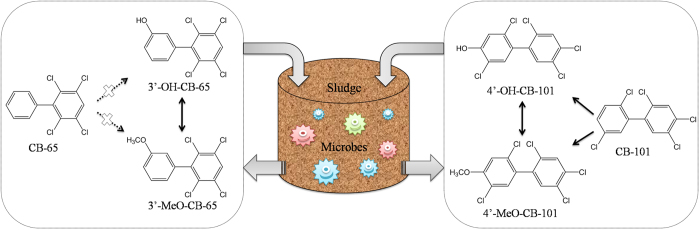
Metabolic results of CB-65 and CB-101, with the homologous OH-PCBs and MeO-PCBs by the microbes in the sludge.

None of the metabolites were found in the blank control, water control or sterile control, suggesting that the microbes in the sludge were responsible for the transformation of PCBs, OH-PCBs, and MeO-PCBs. No cross-contamination was found between the reactors. The purity of the six exposure chemical standards was verified, and there was no undesirable OH-PCB and MeO-PCB detected as impurities that would affect the metabolic results in this study. The metabolic results of PCBs, OH-PCBs, and MeO-PCBs by microbes in sludge are illustrated in Fig. 3. MeO-PCBs may be reaction intermediates in the formation of OH-PCBs from PCBs, making them difficult to detect in the environment. MeO-PCBs may also be the final transformation product of OH-PCBs, though the conversion rate was relatively low.

### Source estimation and environmental implications

Two major reasons for that MeO-PCBs and OH-PCBs were found in the collected sludge samples in this study are as follows: (i) the levels of PCBs were relatively high in some selected WWTPs, such as in Zhejiang Province, Shanghai Municipality, and Guangdong Province; and (ii) abundant and diverse microbes existed in the sewage sludge, which is a special medium and functioned in the entire metabolic process of PCBs, OH-PCBs, and MeO-PCBs. Several Studies show that anaerobic and aerobic processes mediated PCBs degradation. Highly chlorinated PCBs were removed chlorine atoms under anaerobic process and then mineralized under the aerobic condition. The factors influencing the transformation included the complexity of the PCB congener, the type of microorganism employed, and the interaction among the microorganisms^29^,^30^. Our ongoing studies include exploring the key microbe species that are involved in the proposed metabolic pathway in this work.

4′-OH-CB-101 has been found as a major metabolite in various animals^43^,^44^. Although the concentration of 4′-OH-CB-101 was below the detection limit in the influent sample, we could not exclude the possibility that a proportion of 4′-OH-CB-101 might be formed by humans at trace concentration and entered the WWTPs from human excretion. The 4′-MeO-CB-101 could be generated from both CB-101 and 4′-OH-CB-101 by microbes in the sludge. The 4′-MeO-CB-101 was also a potential intermediate that requires further confirmation.

Compared with the conversion rate in the present exposure study, the calculated rates of concentration of both MeO-PCB/PCB and MeO-PCB/OH-PCB were higher in the sludge samples collected from the WWTPs. There are several reasons can explain this. First, the microbial reaction in the WWTPs might be more active than in the laboratory. Second, a portion of the parent compounds in the sewage might be discharged with the effluent without full contact with the sludge. Finally, hydrophobic MeO-PCBs were more easily preserved in the sludge than OH-PCBs. This work was not meant to establish the WWTP sludge as the only source of MeO-PCBs and OH-PCBs; however, it is one source. Previous studies have shown that sludge amendment can be a source of elevated levels of a variety of pollutants to agricultural soils^45^. The presence of OH-PCBs and MeO-PCBs in the sewage sludge may therefore be another cause for concern.

In summary, this is the first study on the detection of MeO-PCBs and OH-PCBs in sewage sludge, which is important because MeO-PCBs are a class of previously undiscovered chemicals in the environment. Microbes in sewage sludge play a key role in the transformation of the PCB analogues, including the hydroxylation and methoxylation of PCBs, as well as the interconversion between OH-PCBs and MeO-PCBs. Wastewater treatment plants are overlooked producers of widespread OH-PCBs in the environment. Other than microbial transformation, the potential sources of OH-PCBs and MeO-PCBs in sewage sludge also include the accumulation from original commercial Aroclors and human excretion. Wastewater treatment plants are a possible emission source of OH-PCBs and MeO-PCBs to the surrounding environment.

## Experimental section

### Materials

The low-chlorinated PCBs (mainly di- to penta-PCBs) are the major PCB homologue group residing in the environment in China^34^. Accordingly, the selected OH-PCB analytes in this study were mainly low-chlorinated congeners, which have been found in sediment samples and original commercial Aroclors at relatively high concentrations^10^. The homologous MeO-PCBs were also selected as targeted analytes. The full names, abbreviations, and chemical structures for the target compounds are shown in Table S1. Although there are 837 pairs of theoretically possible OH-PCBs and MeO-PCBs, the commercially available homologous OH-PCBs and MeO-PCBs are limited at the time of analysis. The ten standards of OH-PCBs were 2′-OH-CB-12, 4-OH-CB-14, 4′-OH-CB-18, 4′-OH-CB-26, 2′-OH-CB-61, 3′-OH-CB-61, 4′-OH-CB-61, 2′-OH-CB-65, 3′-OH-CB-65, and 4′-OH-CB-101. The ten standards of MeO-PCBs were 2′-MeO-CB-12, 4-MeO-CB-14, 4′-MeO-CB-18, 4′-MeO-CB-26, 2′-MeO-CB-61, 3′-MeO-CB-61, 4′-MeO-CB-61, 2′-MeO-CB-65, 3′-MeO-CB-65, and 4′-MeO-CB-101. The ten studied PCBs were CB-18, 26, 28, 52, 61, 65, 101, 138, 153, and 180. The surrogate standards were 4′-MeO-CB-159 and 4′-OH-CB-159 for the neutral and phenolic chemicals, respectively^10^. Acetonitrile, methyl tert-butyl ether (MTBE), hexane, acetone and dichloromethane (DCM) were of HPLC grade or pesticide grade. Silica gel and anhydrous sodium sulfate were activated in advance. Acidified silica gel was prepared by mixing activated silica (70 g) with concentrated H_2_SO_4_ (30 g). Deionized water (18.2 MΩ) was generated via a Milli-Q system.

### Sample collection

The sampling map and sites are shown in Fig. 1. A total of 12 sewage sludge samples were collected from September 2013 to June 2014 from different WWTPs within 12 provinces and municipalities in Greater China. Detailed information on the WWTP characteristics is provided in Table S2. The freshly digested sludge samples (approximately 1 kg for each sample) from the dewatering process were packed in aluminum foil, sealed in kraft bags, and immediately delivered to a laboratory. The samples were then freeze-dried, homogenized, sieved through a stainless steel 100-mesh sieve and preserved at −20 °C until analysis. Two grams of each sludge sample were used in the determination experiments. The calculations of the concentrations of the targeted compounds in the sludge were based on the dry weight (dw). The influent and effluent water samples (approximately 1 L for each sample) were taken from a WWTP in Zhejiang Province in September 2014, from which a sludge sample was previously collected. These water samples were extracted promptly after centrifugation at 5000 rpm for 10 min, and the entire remaining SPM was also collected for further analysis.

### Laboratory-simulated sludge exposure

Laboratory-based exposure studies were conducted to identify the possible reason for the occurrence of the targeted chemicals in the sewage sludge. Based on the results of the field investigation, two PCB congeners, CB-65 and CB-101, two MeO-PCB congeners, 3′-MeO-CB-65 and 4′-MeO-CB-101, and two OH-PCB congeners, 3′-OH-CB-65 and 4′-OH-CB-101, were selected as the exposure compounds. The six compounds were added separately (10 μg) to 65 mL of laboratory-simulated sewage sludge and mixed thoroughly in a 100 mL brown incubator bottle. Seed sludge (15 mL) was added to each of the incubator bottles as a cosubstrate to begin the digestion^46^. The sewage consisted of yeast extract, meat extract, peptone, urea, (NH_4_)_2_SO_4_, K_2_HPO_4_, CaCl_2_, MgSO_4_, and trace element solution^47^.

The blank control (in the absence of the exposure compounds), water control (exposure compounds only in deionized water), and the sterile control (exposure compounds in fully sterile sludge) were prepared similarly to the exposure groups. Each of the bottles was placed simultaneously on an incubated shaker-table at 35 ± 2 °C that was kept under the same conditions. The total exposure time was 5 days, which was similar to the common sludge retention time in WWTPs. The sludge of the exposure group was sampled at intervals of 1, 2, 3, 4 and 5 days. At the end of the exposure time, the control groups were sampled. The exposure and control groups were prepared in triplicate, including the ones for different time intervals. The sludge was freeze-dried, homogenized, and stored at −20 °C prior to analysis. No targeted compounds existed in the simulated sludge that was used in this study before the exposure experiment.

### Sample pretreatment and analysis

The sample pretreatment and analysis were adapted from the previously reported method^21^. Briefly, the solid sample (sludge and SPM) was spiked with surrogate standards and ultrasonically extracted for 60 min twice using hexane/MTBE (1:1 *v/v*; 40 mL). The extracts were combined and evaporated to dryness and redissolved in 50 mL of DCM. Acidified silica gel (10 g) was added, and the mixture was shaken vigorously for 10 min to remove the lipids. The acidified silica gel was then removed via an anhydrous Na_2_SO_4_ column (15 g). An additional 40 mL of DCM was used to further elute the compounds. A secondary purification cycle was performed following the same operational steps. Then, sulfur was eliminated by the addition of activated copper powder (2 g). The extract was concentrated to dryness and dissolved with 400 μL hexane. A half of the extract was transferred into a vial for subsequent analysis of the PCBs and MeO-PCBs by gas chromatography/mass spectrometry (GC/MS). The other half of the extract was dried under a nitrogen stream and redissolved in 200 μL of acetonitrile for analyzing OH-PCBs with liquid chromatography/tandem mass spectrometry (LC/MS/MS). Without the commonly used prior derivatization of OH-PCBs^17^, the entire sample preparation was simplified with satisfying sensitivity of the method.

The water samples were extracted using a liquid–liquid extraction method. The water sample (1L) was spiked with surrogates, mixed with 100 mL of DCM, and shaken for 10 min. Then, the DCM was transferred to another glass bottle, and the extraction was repeated twice. The combined extract was concentrated by rotary evaporation to 50 mL, and further purified and analyzed as described for the solid samples.

The quantitative analysis of the PCBs and MeO-PCBs was conducted on a 7890B/5977A GC/MS instrument (Agilent Technologies, Santa Clara, CA, USA) operated with electron impact source. The GC was fitted with a DB-5 MS capillary column (30 m, 0.25 mm i.d., 0.25 μm film thickness; J&W Scientific, Folsom, CA, USA) with helium as the carrier gas at a constant flow rate of 1.0 mL/min. The oven temperature was initially set at 80 °C, ramped to 140 °C at 10 °C/min, and increased to 300 °C at 2.5 °C/min. The selected ion monitoring (SIM) mode was used for the quantitative determination. The ions that were used to analyze the targeted MeO-PCBs and PCBs are listed in Table S3. The GC/MS chromatograms of PCB and MeO-PCB standards were presented in Figures S1 and S2. A GC with tandem MS (GC/MS/MS) (Agilent 7890B–7000C) was employed to further confirm the identification accuracy of MeO-PCBs. The precursor and product ions of the targeted MeO-PCBs are listed in Table S4.

The quantification of the OH-PCBs was performed on an Agilent 1260–6460 LC/MS/MS instrument. A C18 column (100 mm × 2.1 mm, 2.2 μm particle size, Thermo Fisher Scientific, Waltham, MA, USA) was chosen for chromatographic separation and quantification. The mobile phase consisted of acetonitrile and water, which was used with a gradient elution of a ratio ranging from 45:55 to 90:10 over 35 min at a flow rate of 0.3 mL/min. The MS was operated with a negative electrospray ionization (ESI) source in multiple-reaction monitoring (MRM) mode. Detailed information on the ion transitions that were monitored for each OH-PCB is provided in Table S5. The LC/MS/MS chromatograms of OH-PCB standards were presented in Figure S3. Another Agilent ZORBAX SB-C18 column (150 mm × 2.1 mm, 3.5 μm particle size) was used for further identification of the analytes. The corresponding mobile phase consisted of mehanol and water with a gradient elution of a ratio ranging from 55:45 to 85:15 over 50 min at a flow rate of 0.3 mL/min.

### Quality assurance and quality control

All of the reported data were subject to strict quality assurance and control procedures. No mutual interference was observed in the instrumental analysis of the phenolic and neutral compounds. A procedural blank, a spiked blank, and a sample duplicate were processed in parallel with each batch of six samples. The procedural blank, Na_2_SO_4_, was used to monitor for background contamination levels, and all analytes were under the detection limits. The average recoveries of the PCBs, MeO-PCBs, and OH-PCBs in the spiked samples were 81.3–90.9%, 80.5–91.4% and 73.2–104.2%, respectively, where the relative standard deviation (RSD) was lower than 18% (*n* = 3). The recoveries of the surrogate standards were 85.1–96.4% for 4′-MeO-CB-159, and 83.5–102.2% for 4′-OH-CB-159. Duplicates were included in the sludge sample analysis, and the RSD of the detected concentration was lower than 15% (*n* = 3). The instrumental calibration was verified by injecting five calibration standards, and the linearity of the calibration curve (*R*^2^) was > 0.99. The method limits of detection (MLODs) were calculated at a signal-to-noise ratio of 3. The MLODs for the PCBs, MeO-PCBs and OH-PCBs in the solid samples were 0.2–1.5, 0.1–1.2, and 0.05–0.8 ng/g, respectively. The MLODs for the three groups of compounds in water were 45–180, 60–200, and 20–80 pg/L, respectively. The statistical analysis including the Pearson’s correlation analysis was performed using SPSS 18.0 and Origin 8.0. Statistical significance was considered as *p* < 0.05.

## Additional Information

**How to cite this article**: Sun, J. *et al*. Detection of methoxylated and hydroxylated polychlorinated biphenyls in sewage sludge in China with evidence for their microbial transformation. *Sci. Rep.* **6**, 29782; doi: 10.1038/srep29782 (2016).

## Supplementary Material

Supplementary Information

## Figures and Tables

**Table 1:** Concentrations (ng/g, dry weight) of PCBs, OH-PCBs, and MeO-PCBs in the sewage sludge collected in the investigated wastewater treatment plants in Greater China.

**Compound**	**Mean**	**Min.**	**Max.**	**Detection rate (%)**
PCB-18	nd^a^	nd	nd	0
PCB-26	nd	nd	nd	0
PCB-28	15.2	1.4	63.1	100
PCB-52	9.68	0.71	50.8	100
PCB-61	nd	nd	nd	0
PCB-65	nd	nd	nd	0
PCB-101	6.40	0.74	35.2	100
PCB-138	2.71	nd	11.5	83
PCB-153	1.82	nd	6.00	75
PCB-180	2.58	nd	3.40	33
∑10 PCBs	35.8	3.04	170	100
2′-OH-CB-12	nd	nd	nd	0
4-OH-CB-14	nd	nd	nd	0
4′-OH-CB-18	0.52	nd	0.62	42
4′-OH-CB-26	0.57	nd	0.73	33
2′-OH-CB-61	nd	nd	nd	0
3′-OH-CB-61	nd	nd	nd	0
4′-OH-CB-61	nd	nd	nd	0
2′-OH-CB-65	nd	nd	nd	0
3′-OH-CB-65	2.13	nd	4.20	58
4′-OH-CB-101	2.82	nd	6.20	42
∑10 OH-PCBs	4.23	nd	11.5	67
2′-MeO-CB-12	nd	nd	nd	0
4-MeO-CB-14	nd	nd	nd	0
4′-MeO-CB-18	nd	nd	nd	0
4′-MeO-CB-26	nd	nd	nd	0
2′-MeO-CB-61	nd	nd	nd	0
3′-MeO-CB-61	nd	nd	nd	0
4′-MeO-CB-61	nd	nd	nd	0
2′-MeO-CB-65	nd	nd	nd	0
3′-MeO-CB-65	0.58	nd	0.89	25
4′-MeO-CB-101	0.52	nd	0.65	25
∑10 MeO-PCBs	1.10	nd	1.54	25

^a^Nondetectable.

**Table 2:** Detection of metabolites after exposure of the sludge for 5 days, and their rates of concentration in WWTP sludge.

**Exposure compound**	**Metabolite**	**Conversion**^a^ **(%)**	**Rates in WWTP sludge**
CB-65	3′-OH-CB-65	nd^b^	nd
CB-65	3′-MeO-CB-65	nd	nd
3′-OH-CB-65	3′-MeO-CB-65	1.51 ± 0.22	0.15 ± 0.06
3′-MeO-CB-65	3′-OH-CB-65	6.83 ± 0.88	7.39 ± 2.81
CB-101	4′-OH-CB-101	1.65 ± 0.21	0.22 ± 0.05
CB-101	4′-MeO-CB-101	0.40 ± 0.08	0.03 ± 0.01
4′-OH-CB-101	4′-MeO-CB-101	1.75 ± 0.19	0.12 ± 0.02
4′-MeO-CB-101	4′-OH-CB-101	9.47 ± 1.04	8.28 ± 1.30

^a^Mean ± standard deviation (*n* = 3).

^b^Nondetectable.
