# Supplementary material for: Detection of methoxylated and hydroxylated polychlorinated biphenyls in sewage sludge in China with evidence for their microbial transformation
Source: Sci Rep. 2016 Jul 15;6:29782. doi: 10.1038/srep29782 (PMC4945941; doi:10.1038/srep29782)
Supplement: Supplementary Information [file srep29782-s1.doc]

**Supporting Information**

**Detection of methoxylated and hydroxylated** **polychlorinated biphenyls in** **sewage sludge in China with evidence for their microbial transformation**

**Jianteng Sun^1, 2^, Lizhong Zhu^1, 2, *^, Lili Pan^1^, Zi Wei^3^, Yao Song^1^, Yuduo Zhang^1^, Liping Qu^4^ & Yu Zhan^1^**

^1^Department of Environmental Science, Zhejiang University, Hangzhou, Zhejiang 310058, China

^2^Zhejiang Provincial Key Laboratory of Organic Pollution Process and Control, Hangzhou, Zhejiang 310058, China

^3^Analysis and Measurement Center, Zhejiang University, Hangzhou, Zhejiang 310058, China

^4^THUNIP Holdings Co., Ltd., Beijing 100020, China

^*^ Corresponding author: Lizhong Zhu (L.Z.)

Phone/fax: +86 57188273733

E-mail: [zlz@zju.edu.cn](mailto:zlz@zju.edu.cn)

**Contents**

**Table S1.** Full names, abbreviations, and chemical structures for the targeted compounds

**Table S2.** Detailed information on the investigated WWTPs

**Table S3.** Monitoring ions for analyzing the targeted MeO-PCBs and PCBs by GC/MS

**Table S4.** Precursor and product ions of the targeted MeO-PCBs by GC/MS/MS

**Table S5.** Monitored MRM transitions for the determination of OH-PCB congeners

**Figure S1.** GC/MS chromatograms of PCB standards mixture: (1) PCB-18; (2) PCB-26; (3) PCB-28; (4) PCB-52; (5) PCB-65; (6) PCB-61; (7) PCB-101; (8) PCB-153; (9) PCB-138; (10) PCB-180

**Figure S2.** GC/MS chromatograms of MeO-PCB standards mixture: (1) 4-MeO-CB-14; (2) 2’-MeO-CB-12; (3) 4’-MeO-CB-18; (4) 2’-MeO-CB-65; (5) 4’-MeO-CB-26; (6) 3’-MeO-CB-65; (7) 2’-MeO-CB-61; (8) 3’-MeO-CB-61; (9) 4’-MeO-CB-61; (10) 4’-MeO-CB-101; (11) 4’-MeO-CB-159 (surrogate standard)

**Figure S3.** Total ion chromatogram (TIC) and individual MRM chromatograms of OH-PCB standards on C18 column (100 mm × 2.1 mm, 2.2 μm particle size)

| **Full name** | **Abbreviation** | **Chemical structure** |
| --- | --- | --- |
| 2,2’,5-trichlorobiphenyl | CB-18 | 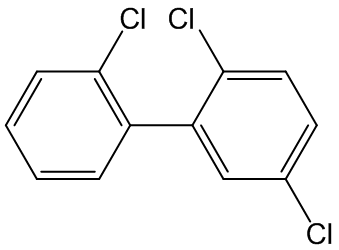 |
| 2,3’,5-trichlorobiphenyl | CB-26 | 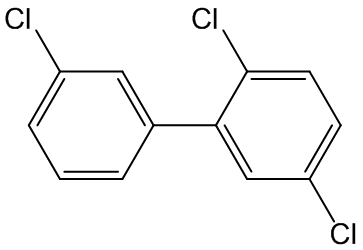 |
| 2,4,4’-trichlorobiphenyl | CB-28 | 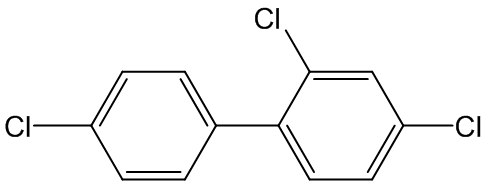 |
| 2,2’,5,5’-tetrachlorobiphenyl | CB-52 | 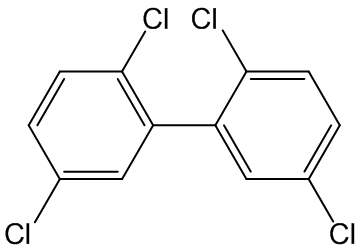 |
| 2,3,4,5-tetrachlorobiphenyl | CB-61 | 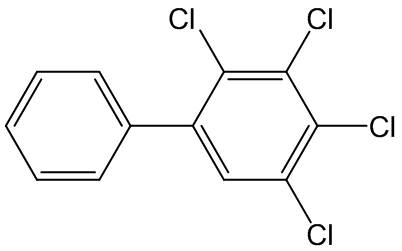 |
| 2,3,5,6-tetrachlorobiphenyl | CB-65 | 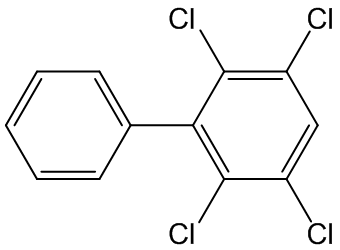 |
| 2,2’,4,5,5’-pentachlorobiphenyl | CB-101 | 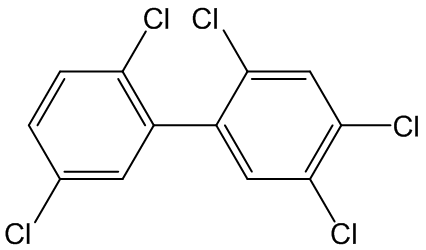 |
| 2,2’,3,4,4’,5’-hexachlorobiphenyl | CB-138 | 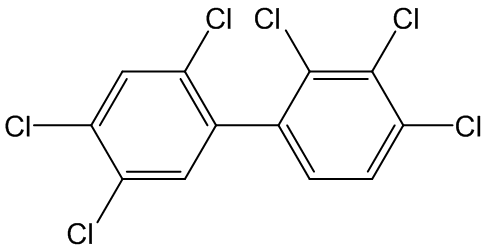 |
| 2,2’,4,4’,5,5’-hexachlorobiphenyl | CB-153 | 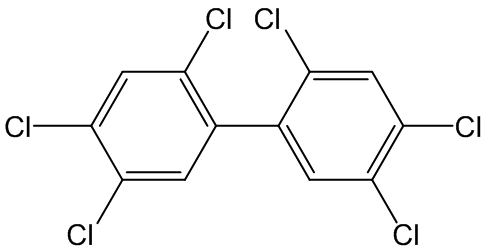 |
| 2,2’,3,4,4’,5,5’-heptachlorobiphenyl | CB-180 | 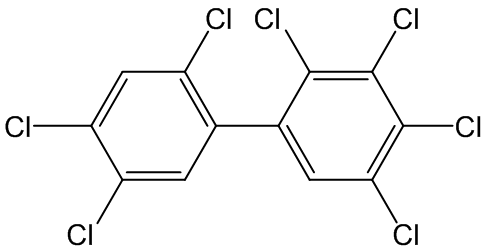 |
| 2’-hydroxy-3,4-dichlorobiphenyl | 2’-OH-CB-12 | 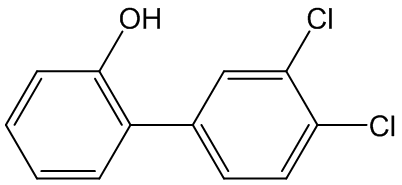 |
| 4-hydroxy-3,5-dichlorobiphenyl | 4-OH-CB-14 | 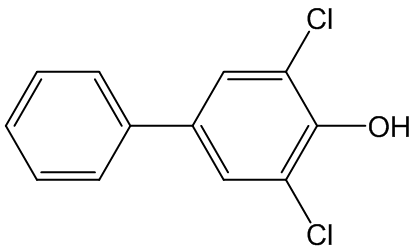 |
| 4’-hydroxy-2,2’,5-trichlorobiphenyl | 4’-OH-CB-18 | 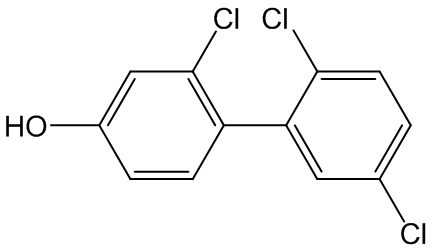 |
| 4’-hydroxy-2,3’,5-trichlorobiphenyl | 4’-OH-CB-26 | 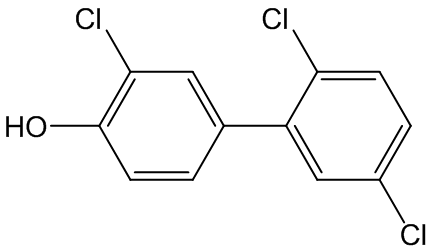 |
| 2’-hydroxy-2,3,4,5-tetrachlorobiphenyl | 2’-OH-CB-61 | 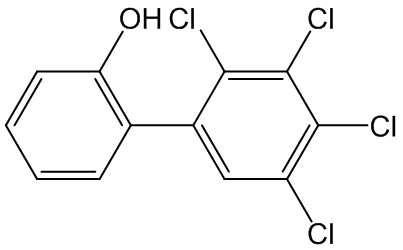 |
| 3’-hydroxy-2,3,4,5-tetrachlorobiphenyl | 3’-OH-CB-61 | 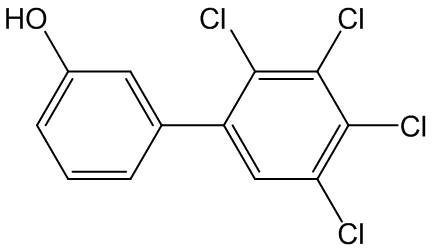 |
| 4’-hydroxy-2,3,4,5-tetrachlorobiphenyl | 4’-OH-CB-61 | 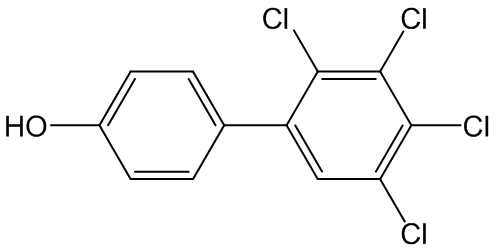 |
| 2’-hydroxy-2,3,5,6-tetrachlorobiphenyl | 2’-OH-CB-65 | 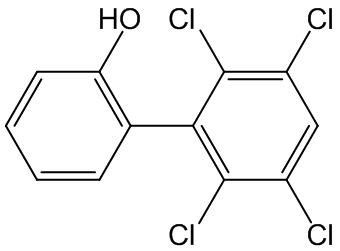 |
| 3’-hydroxy-2,3,5,6-tetrachlorobiphenyl | 3’-OH-CB-65 | 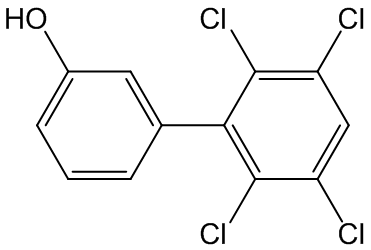 |
| 4’-hydroxy-2,2’,4,5,5’-pentachlorobiphenyl | 4’-OH-CB-101 | 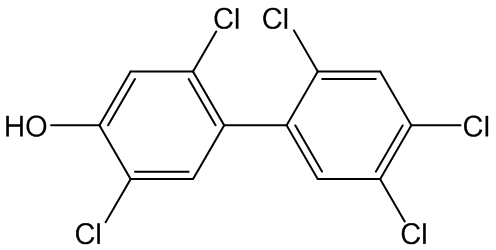 |
| 2’-methoxy-3,4-dichlorobiphenyl | 2’-MeO-CB-12 | 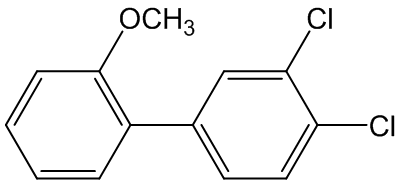 |
| 4-methoxy-3,5-dichlorobiphenyl | 4-MeO-CB-14 | 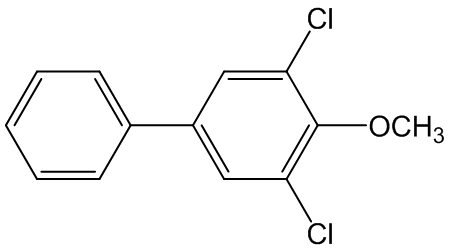 |
| 4’-methoxy-2,2’,5-trichlorobiphenyl | 4’-MeO-CB-18 | 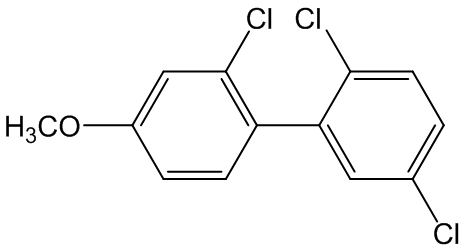 |
| 4’-methoxy-2,3’,5-trichlorobiphenyl | 4’-MeO-CB-26 | 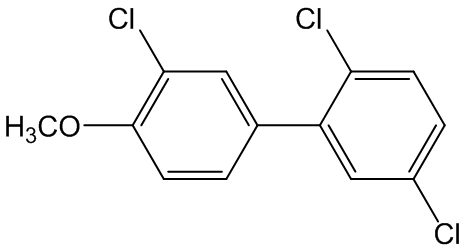 |
| 2’-methoxy-2,3,4,5-tetrachlorobiphenyl | 2’-MeO-CB-61 | 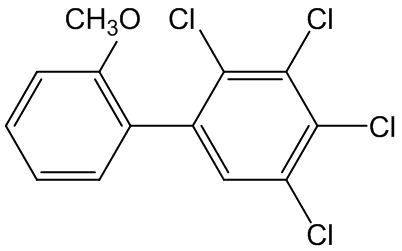 |
| 3’-methoxy-2,3,4,5-tetrachlorobiphenyl | 3’-MeO-CB-61 | 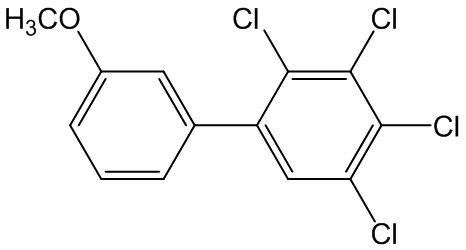 |
| 4’-methoxy-2,3,4,5-tetrachlorobiphenyl | 4’-MeO-CB-61 | 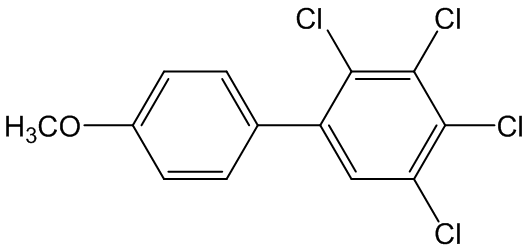 |
| 2’-methoxy-2,3,5,6-tetrachlorobiphenyl | 2’-MeO-CB-65 | 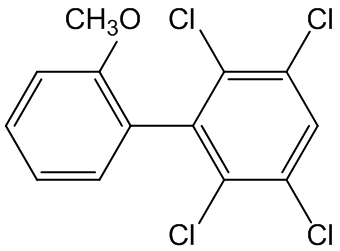 |
| 3’-methoxy-2,3,5,6-tetrachlorobiphenyl | 3’-MeO-CB-65 | 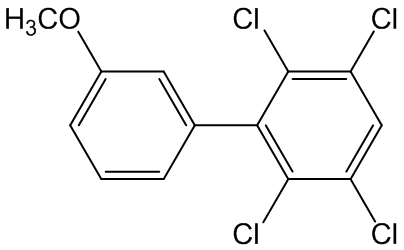 |
| 4’-methoxy-2,2’,4,5,5’-pentachlorobiphenyl | 4’-MeO-CB-101 | 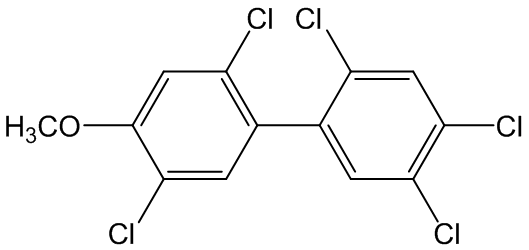 |

**Table S1. Full Names, abbreviations, and chemical structures for the targeted Compounds.**

| **Sampling location** | **Processing volume**  **(10^4^ m^3^/d)** | **Biotreatment**  **techniques *^a^*** | **TOC**  **(%)** |
| --- | --- | --- | --- |
| Heilongjiang | 20 | AAO | 35.5 |
| Beijing | 10 | AO | 37.4 |
| Xinjiang | 4.0 | OD | 25.8 |
| Qinghai | 2.0 | OD | 30.8 |
| Shaanxi | 20 | AAO | 29.9 |
| Zhejiang | 4.0 | AAO | 16.5 |
| Shanghai | 6.0 | AO | 24.6 |
| Guangdong | 10 | OD | 33.4 |
| Sichuan | 3.0 | SBR | 40.2 |
| Hubei | 15 | AAO | 23.6 |
| Yunnan | 5.0 | AO | 16.3 |
| Henan | 7.0 | AO | 20.6 |
| **Table S2. Detailed information on the investigated WWTPs.** *^a^*WWTP activated sludge biotreatment techniques. AAO: anaerobic-anoxic-oxic process, OD: oxidation ditch process, AO: anoxic/oxic process, SBR: sequencing batch reactor process. | | | |

| **Compound** | **Formula** | **Retention time (min)** | **Quantitative ion**  ***m/z* 1** | **Qualitative ion**  ***m/z* 2** | |
| --- | --- | --- | --- | --- | --- |
| CB-18 | C_12_H_7_Cl_3_ | 17.390 | 256 | 186 |  |
| CB-26 | C_12_H_7_Cl_3_ | 19.702 | 256 | 186 |  |
| CB-28 | C_12_H_7_Cl_3_ | 20.290 | 256 | 186 |  |
| CB-52 | C_12_H_6_Cl_4_ | 22.568 | 292 | 220 |  |
| CB-61 | C_12_H_6_Cl_4_ | 26.262 | 292 | 220 |  |
| CB-65 | C_12_H_6_Cl_4_ | 23.203 | 292 | 220 |  |
| CB-101 | C_12_H_5_Cl_5_ | 28.435 | 326 | 254 |  |
| CB-138 | C_12_H_4_Cl_6_ | 36.083 | 360 | 290 |  |
| CB-153 | C_12_H_4_Cl_6_ | 34.267 | 360 | 290 |  |
| CB-180 | C_12_H_3_Cl_7_ | 40.730 | 394 | 324 |  |
| 2’-MeO-CB-12 | C_13_H_10_OCl_2_ | 22.935 | 252 | 211 |  |
| 4-MeO-CB-14 | C_13_H_10_OCl_2_ | 22.262 | 252 | 209 |  |
| 4’-MeO-CB-18 | C_13_H_9_OCl_3_ | 26.013 | 288 | 245 |  |
| 4’-MeO-CB-26 | C_13_H_9_OCl_3_ | 29.541 | 288 | 245 |  |
| 2’-MeO-CB-61 | C_13_H_8_OCl_4_ | 31.475 | 322 | 279 |  |
| 3’-MeO-CB-61 | C_13_H_8_OCl_4_ | 34.411 | 322 | 279 |  |
| 4’-MeO-CB-61 | C_13_H_8_OCl_4_ | 35.866 | 322 | 279 |  |
| 2’-MeO-CB-65 | C_13_H_8_OCl_4_ | 28.131 | 322 | 279 |  |
| 3’-MeO-CB-65 | C_13_H_8_OCl_4_ | 31.117 | 322 | 279 |  |
| 4’-MeO-CB-101 | C_13_H_7_OCl_5_ | 37.508 | 356 | 313 |  |

**Table S3. Monitoring ions for analyzing the targeted MeO-PCBs and PCBs by GC/MS.**

| **Compound** | **Retention time**  **(min)** | **Precursor**  ***m/z*** | **Product**  ***m/z*** |
| --- | --- | --- | --- |
| 2’-MeO-CB-12 | 24.957 | 252.2 | 202.2 |
| 4-MeO-CB-14 | 24.258 | 252.2 | 209.2 |
| 4’-MeO-CB-18 | 28.037 | 287.5 | 245 |
| 4’-MeO-CB-26 | 31.745 | 287.5 | 245 |
| 2’-MeO-CB-61 | 33.498 | 322 | 272 |
| 3’-MeO-CB-61 | 36.565 | 322 | 221.8 |
| 4’-MeO-CB-61 | 38.117 | 322 | 278.8 |
| 2’-MeO-CB-65 | 30.090 | 322 | 272 |
| 3’-MeO-CB-65 | 33.144 | 322 | 221.8 |
| 4’-MeO-CB-101 | 39.574 | 356.4 | 312.9 |

**Table S4. Precursor and product ions of the targeted MeO-PCBs by GC/MS/MS.**

| **Compound** | **Formula** | **Retention time (min)** | | **MRM transition**  **(m/z)** | **Frag**  **(V)** | **CE**  **(V)** |
| --- | --- | --- | --- | --- | --- | --- |
|  |  | **Column A^a^** | **Column B^b^** |  |  |  |
| 2’-OH-CB-12 | C_12_H_8_OCl_2_ | 8.857 | 21.859 | 237→35.1 | −103 | −24 |
| 4-OH-CB-14 | C_12_H_8_OCl_2_ | 8.307 | 20.713 | 237→35.1 | −103 | −24 |
| 4’-OH-CB-18 | C_12_H_7_OCl_3_ | 8.857 | 21.859 | 270.9→35.1 | −103 | −24 |
| 4’-OH-CB-26 | C_12_H_7_OCl_3_ | 10.074 | 25.518 | 270.9→35.1 | −98 | −24 |
| 2’-OH-CB-61 | C_12_H_6_OCl_4_ | 13.12 | 32.906 | 305→35.1 | −70 | −4 |
| 3’-OH-CB-61 | C_12_H_6_OCl_4_ | 13.646 | 35.034 | 305→268.9 | −137 | −16 |
| 4’-OH-CB-61 | C_12_H_6_OCl_4_ | 14.109 | 36.369 | 304.9→35.1 | −101 | −28 |
| 2’-OH-CB-65 | C_12_H_6_OCl_4_ | 11.189 | 27.269 | 304.9→35.1 | −70 | −4 |
| 3’-OH-CB-65 | C_12_H_6_OCl_4_ | 11.487 | 28.996 | 304.9→35.1  304.9→268.9^c^ | −122  −122 | −32  −16 |
| 4’-OH-CB-101 | C_12_H_5_OCl_5_ | 16.465 | 40.043 | 338.9→35.1  338.9→302.9^c^ | −101  −101 | −28  −24 |

**Table S5. Monitored MRM transitions for the determination of OH-PCB congeners.** ^a^Column A: Thermo Fisher Scientific Acclaim RSLC 120 C18 column (100 mm × 2.1 mm, 2.2 μm particle size). ^b^Column B: Agilent ZORBAX SB-C18 column (150 mm × 2.1 mm, 3.5 μm particle size). ^c^MRM transition used for further identification

**
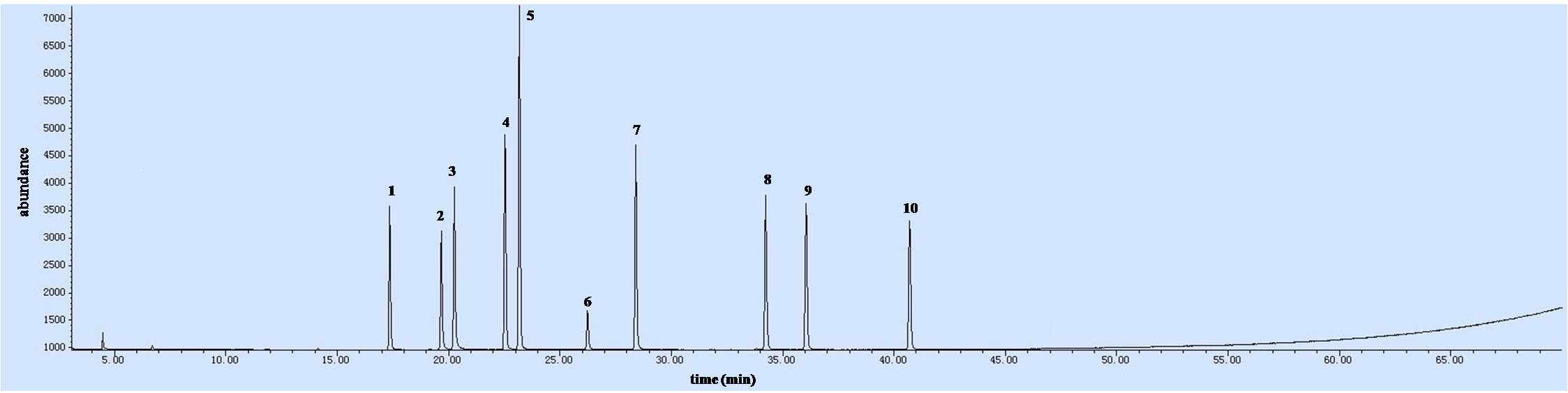
**

**Figure S1. GC/MS chromatograms of PCB standards mixture: (1) PCB-18; (2) PCB-26; (3) PCB-28; (4) PCB-52; (5) PCB-65; (6) PCB-61; (7) PCB-101; (8) PCB-153; (9) PCB-138; (10) PCB-180.**


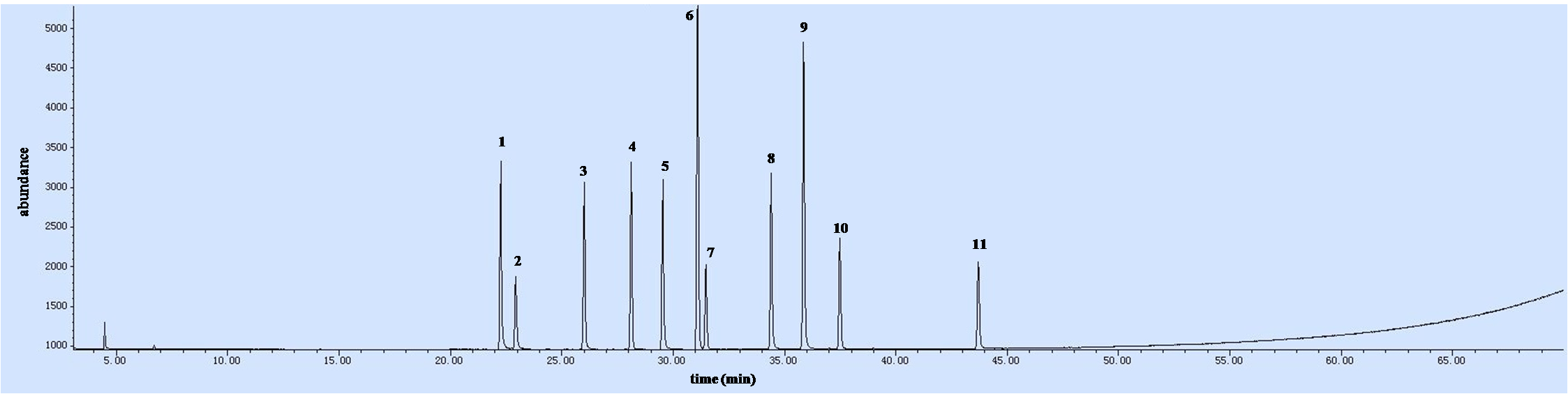


**Figure S2. GC/MS chromatograms of MeO-PCB standards mixture: (1) 4-MeO-CB-14; (2) 2’-MeO-CB-12; (3) 4’-MeO-CB-18; (4) 2’-MeO-CB-65; (5) 4’-MeO-CB-26; (6) 3’-MeO-CB-65; (7) 2’-MeO-CB-61; (8) 3’-MeO-CB-61; (9) 4’-MeO-CB-61; (10) 4’-MeO-CB-101; (11) 4’-MeO-CB-159 (surrogate standard).**


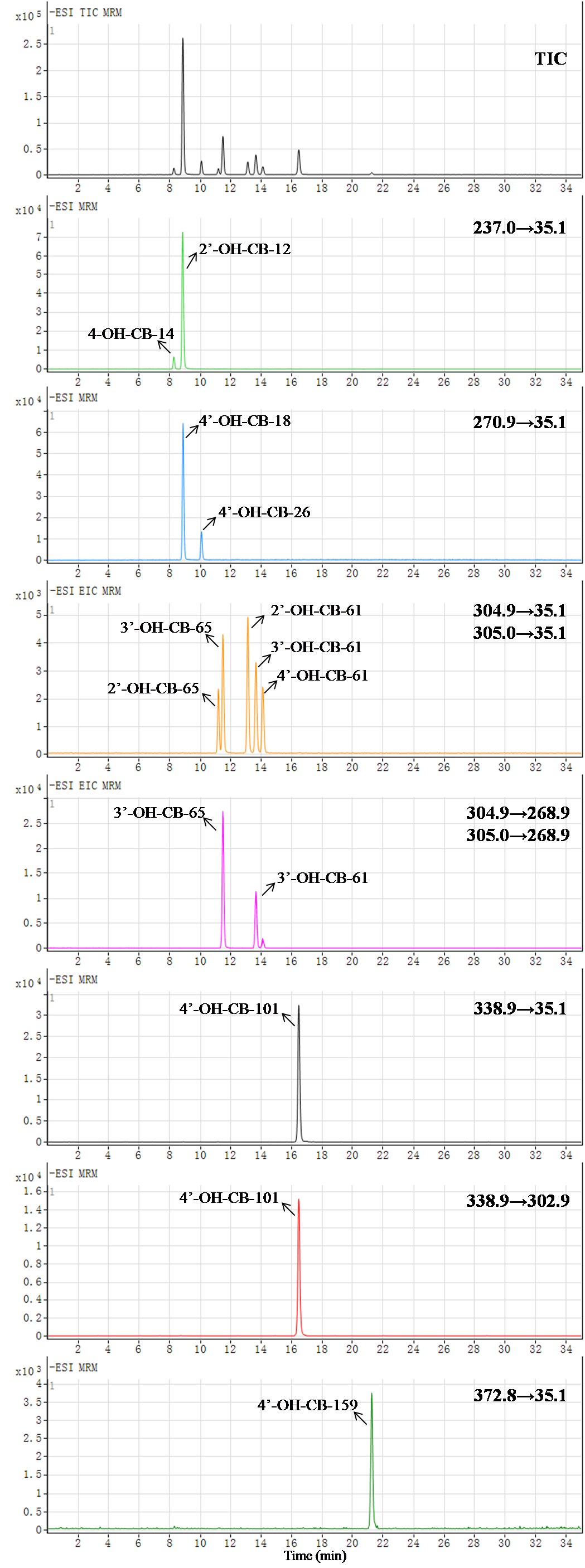


**Figure S3. Total ion chromatogram (TIC) and individual MRM chromatograms of OH-PCB standards on C18 column (100 mm × 2.1 mm, 2.2 μm particle size).**
